# The impact of COVID-19 pandemic on food habits and neophobia in children in the framework of the family context and parents’ behaviors: A study in an Italian central region

**DOI:** 10.3389/fnut.2022.1070388

**Published:** 2022-12-08

**Authors:** Annalisa Di Nucci, Umberto Scognamiglio, Federica Grant, Laura Rossi

**Affiliations:** Research Centre for Food and Nutrition, Council for Agricultural Research and Economics (CREA), Rome, Italy

**Keywords:** neophobia, eating habits, COVID-19 restrictions, children, Italy

## Abstract

**Objective:**

This paper aims to evaluate whether changes in lifestyle and eating habits resulting from the Covid-19 emergency have influenced the post-pandemic level of food neophobia and in children living in an Italian central region.

**Methods:**

A sample of 99 children took part in a retrospective assessment carried out with a self-administrated questionnaire. Pre and post-pandemic evaluation of eating habits, physical activity, and lifestyle indicators was carried out. Food neophobia was evaluated following the Child Food Neophobia Scale (CFNS). Descriptive statistics were produced. A contingency analysis was performed to check associations between variables.

**Results:**

For a large part of the sample (97%) the selective food refusal did not change during the pandemic period. About 70% of participants did not change their eating habits, with some subgroups reporting an increase in the consumption of fruits (22.2%), vegetables (19.2%), and legumes (21.2%). Relevantly the impact of the pandemic on the sedentary attitude passed from 25.3 to 70.7%. Neophobia was not associated with ponderal status (*p*-value 0.5). However, in normal-weight children, a high prevalence of intermediate-level neophobia (78.4%) was found. 39.4% of the studied children were involved in meal preparation during social isolation, with an increase in the proportion of children that shared all meals with their family (32.3% vs. 78.8%). Non-coercive parent behaviors in reaction to food refusal were associated with low levels of neophobia (*p*-value < 0.05).

**Discussion:**

In this sample, for the effect of parents’ attitudes, the pandemic positively affected children’s food habits and, consequently, the level of neophobia after the social restrictions. The main implication of the study is the importance of capitalizing on the period of restrictions in order to involve children in meal sharing and food preparation.

## Introduction

In children of the Mediterranean region, there has been a trend of abandoning the Mediterranean Diet (MD) with a shift toward a Westernized dietary pattern (1). In Italy, data collected in 2019 (2) highlighted that the consumption of fruit, vegetables, and legumes, typical MD foods, was inadequate for many children.

One of the causes of low fruit and vegetable consumption in children could be food neophobia which is defined as the reluctance to eat new or unknown foods. It is a very common behavior among children with a well-defined onset and evolution. Normally neophobia appears during the complementary feeding period, at 4–6 months of age when food is gradually introduced into the child’s diet, it increases sharply as the child becomes more mobile and independent, reaching a peak between 2 and 6 years of age and then gradually decreases into adulthood (3).

As reported by Cooke et al. (4), food neophobia is primarily a hereditary trait, in which the genetic determinants accounted for 78%. However, the development of eating behavior is determined by the dynamic interplay of genetics (5), environmental factors (e.g., interaction with caregiver) (6) and food-related experiences (7). Among environmental factors, the so-called social facilitation mechanism (8) should be mentioned, which is characterized by an improvement in the performance of a task in the presence of others, e.g., family components, compared to behavior when staying alone. Translating this concept to food habits, the more the people around a child consume new and unusual food, the more willing the child will be to try it (3). It has been observed that how food is offered to the child has a significant impact on the development of food neophobia. Parental pressure for children to eat foods they do not like results in greater resistance to consumption. In addition, the absence of affectionate behavior during meals results in children associating eating with negative emotions such as anxiety and tension. These emotions reinforce the rejection of unfamiliar foods when offered and exacerbate neophobic behaviors (9).

From an evolutionary point of view, food neophobia could be considered a protective mechanism that reduces the risk of eating potentially harmful foods. Reinforcing the habit of choosing familiar and safe foods, instead of new, unfamiliar, and potentially dangerous food (10). For these reasons, the neophobic attitude is mainly manifested in the consumption of foods with bitter or acidic tastes such as fruits and vegetables, in which it is more likely to find unsafe substances (11–14). As well as manifesting in the case of animal foods which are primary sources of bacteria responsible for food toxicoinfections (15).

It was also demonstrated that food neophobia limits the adherence to the MD in the sense that a higher level of food neophobia is associated with lower adherence to the MD (16).

Food neophobia does not limit the amount of ingested food but impacts the variety and nutritional quality of the diet (17). The neophobic people’s diets are often characterized by a high intake of saturated fats and sugars (17, 18), foods not targeted by neophobia considering the innate preference for sweet and savory flavors (13, 19). This dietary pattern limits the intake of several nutrients such as vitamin E, folate, calcium, zinc, and fibers that are essential, especially in childhood, for physical and intellectual development and to prevent future occurrence of chronic non-communicable diseases (9).

For these reasons, it was hypothesized that food neophobia could be a predictor of childhood obesity as parents compensate for children’s reluctance to eat healthy foods by providing foods that are more accepted such as sweet and calorie-dense foods (18, 20). However, limited studies are available on the relationship between food neophobia and child weight status, and the findings are not univocal. Absence of association between food neophobia and weight status was frequently reported (19, 21–23), however, an increase of overweight related to neophobia was also observed (24). According to Rioux (25), to date there are insufficient studies demonstrating a robust association between food neophobia and child weight status.

The covid-19 pandemic changed people’s daily behaviors including lifestyle and eating habits. Due to the rapid spread of the virus in China and other countries around the world, on the 30th January 2020, the World Health Organization (WHO) Emergency Committee declared the Covid-19 disease a global health emergency (26), and then, on 11 March 2020, a pandemic disease (27). Consequently, to contain the spread of the virus, the Italian government approved a series of rigorous containment measures which consisted of an intense social isolation (28). As an effect of these containment measures children could not attend school, see their friends, or play sports activities, and this negatively impacted their psychological and physical health status (29). Several Italian studies on children’s lifestyles and eating habits during the first lockdown showed a drastic reduction of time spent in physical activity and more time dedicated to sedentary activities such as watching TV and playing video games (30–32). This could explain the weight gain observed during the COVID-19 pandemic, especially in children already suffering from overweight (33). In addition to that, studies carried out during the severe phases of the pandemic reported a worsening of eating habits characterized by increased consumption of calorie-dense and comfort foods such as chocolate, sweet snacks, and desserts but also bread, pizza, and bakery products (28, 30, 34). The increased consumption of these foods could be related to anxiety (35) attributable to the disruption of the daily routine, limitation of physical activities and opportunities for socialization (36) but also to fight against boredom resulting from the long time spent at home during social isolation (37, 38).

However, the lockdown also had positive effects, creating opportunities to involve children in the preparation of meals (31, 32) and to consume foods with the rest of the family (32), contributing positively to the conviviality and familiarization with foods they do not like (32, 39).

In consideration of the present scenario, we decided to assess the impact of social restrictions related to the Covid-19 pandemic on eating habits and the occurrence of neophobia in children living in Lazio, an Italian central region. The hypothesis underlying this study was that several conditions related to the social restrictions of the pandemic influenced the children’s neophobic behavior. Stress, worries, and bad mood caused by the COVID-19 pandemic may have affected parents’ attitudes toward foods, for example forcing the child to eat, showing disapproval, or using food as a reward, all conditions that promote the neophobic attitude. In consideration of this hypothesis, the main purpose of this study was to evaluate whether changes in lifestyle and eating habits resulting from the Covid-19 emergency have influenced the post—pandemic food neophobia level in a sample of children. Specific objectives of the experimental work were the identification of the factors promoting or attenuating the neophobic attitude as well as the analysis of the strategies able to counteract neophobic behavior and promote healthy eating habits in children.

This study would contribute to addressing the following research questions: (i) to what extent and in which sense did the social restrictions related to the Covid-19 pandemic influence the neophobic attitude? (ii) could there be unexpected positive effects of the Covid-19 lockdown on children’s neophobia? (iii) what were the lessons learned from this extraordinary experience that could be capitalized on in other contexts?

## Materials and methods

### Study design

The present assessment is a retrospective study carried out on a sample of children aged 2–11 years and living in the Italian central region of Lazio. Pre and post—pandemic evaluation of eating habits, physical activity, and lifestyle indicators was carried out and the reported changes were considered in the light of the level of neophobia of studied children measured at the time of the assessment. The class of age and the geographical provenience were the eligibility criteria of the study that consisted of an online administration of a questionnaire distributed through instant messaging apps (e.g., WhatsApp), and by mailing personal contacts. Google Form^®^ was used for online data collection for a self-reported compilation. This simple non-probabilistic approach to recruiting respondents online, by inviting them to follow a link to a survey sent by email, or other similar means, is defined as “river” sampling by Lehdonvirta et al. (40). A sample of 106 children was reached at the end of the survey period. The data collection was performed between July 26 and October 1, 2021, and the answers referred to the conditions related to the second lockdown that started in Italy on November 6, 2020 (41).

### Data collection procedure

Following the European Commission General Data Protection Regulation (679/2016) those willing to participate signed a privacy policy and consent form concerning the collection and processing of socio-demographic data in advance. Before starting the data collection, participants were informed about the objective of the research, the consequent statistical analysis, and the intention to publish the results of the assessment in a scientific journal. Participation in the study was fully voluntary and anonymous and subjects could withdraw from the study at any time for any reason. This study was conducted according to the guidelines of the Declaration of Helsinki (42). The present research is not considered either as medical experimentation, or a direct intervention on human subjects with diet changes or formulated food administration and did not involve any invasive procedures. In addition to that the Council for the Research Economics and Agriculture (CREA) is part of the National Statistical System (SISTAN) and guarantees individual data protection (43). Hence an additional ethical committee review of the study protocol was considered unnecessary once informed consent was obtained.

### The questionnaire

The questionnaire was specifically developed for the scope of the survey and the respondent was an adult that acted as a caregiver. A total number of 57 questions were provided in four sections: (1) socio-demographic data of parents and children (9 questions on age, gender, caregiver’s education, number of family members, presence of children under 11 in the family, weight, and height of the child); (2) Eating habits and lifestyle of the child in the pre-pandemic period (20 questions on eating habits, school catering, the conviviality of the meals, time spent in physical activities, and the use of electronic devices); (3) Eating habits and lifestyle of the child during the second lockdown (18 questions on eating habits, the conviviality of the meals, impact of the covid-19 pandemic on eating habits and eating behavior, caregiver’s feeding practices, time spent in physical activities, and the use of electronic devices); (4) Evaluation of food neophobia.

The questions on eating habits, physical activity, and lifestyle were taken from the National Statistics Multi-purpose Survey on Families: Aspects of Daily Life (44) and from the surveillance system on overweight and obesity and related risk factors carried out every 2 years in children of primary schools (45). These questions were used in other studies carried out in Italy (46–48). Food Neophobia was quantified with the Child Food Neophobia Scale (CFNS) developed by Pliner (49) and validated in Italy by Laureati et al. (50). The CFNS consists of 10 items (five referring to neophiliac and five to neophobic attitude) evaluated with a 7-point scale ranging from 1 = “I strongly disagree” to 7 = “I strongly agree.” The full questionnaire is reported in Supplementary Table 1.

Eating habits data were compared with the current Italian recommendations as reported in the Italian Dietary Guidelines—IDGs (51). Physical activity level was compared with the WHO guidelines that recommend at least an average of 60 min per day of moderate-to-vigorous intensity, mostly aerobic, physical activity, across the week (52). The inactivity level was evaluated in consideration of advice from the Italian Society of Pediatrics that recommend no more than 1 h per day of sedentary activities in children aged 2–5 years and no more than 2 h per day in children aged 5–8 years (53).

The individual CFNS scores were computed according to Predieri et al. (16) as the sum of the scores of the 10 items, reversing the neophiliac items to have a univocal sense of all the responses. Therefore, the scores theoretically ranged from 10 to 70 with higher scores reflecting higher Food Neophobia (FN) levels. The frequency distribution of CFNS scores was calculated and respondents were divided into three groups according to their FN level: low, medium, and high. A standardized way to develop cut-offs of FN scores for classifying individuals as neophobic or neophiliac does not exist because this tool examines the neophobia-neophilia continuum in humans (54) and some authors have used the mean value of FN scores as the cut-off point to differentiate between neophobic and neophiliac consumers (55, 56). However, in this study we used the method most commonly applied for neophobic classification that differentiated 3 consumer segments corresponding to neutrals (score in the interval mean ± 1 SD), neophobic (score > mean + 1 SD), and neophiliac (score < mean – 1 SD) (18, 57).

The child’s weight and height were reported by the respondents. Since Body Mass Index (BMI—kg/m^2^) was calculated and then compared with growth charts of the WHO to evaluate weight status based on the cut-offs for evolutive age proposed by WHO (58, 59).

### Statistical analysis

Descriptive statistics of the data collected were produced. Single continuous and categorical variables were summarized as mean ± standard deviation and percentage (%). A contingency analysis was performed to check associations between variables. Specifically, double-entry tables were processed, and the Chi-square test of independence was applied, along with *post-hoc* tests to check pairwise comparisons, with Bonferroni corrections of the *p*-values. The test of independence on the mean was applied to compare continuous variables with categorical variables. Results were considered significant for *p*-value < 0.05. Statistical analysis was performed using Microsoft^®^ Excel software.

## Results

### Socio-demographic characteristics of the sample

The survey was completed by 106 children. After data cleaning, 7 questionnaires were excluded from the analysis for the following reasons: 3 did not meet the inclusion criteria because were out of the classes of age for the study as defined in the inclusion criteria and for the remaining 4 there was an inconsistency between socio-demographic and anthropometric data (parents declared a child’s stature unrealistically with respect to the age). Then the results were based on a sample of 99 children (52.5% males and 47.5% females). With this sample size the precision level of the study was 9%, according to the formula of Pourhoseingholi et al. (60) based on the expected prevalence of Neophobia in Italy; 26% (16). The value for Cronbach’s Alpha for the study was α = 0.60 corresponding to moderate internal consistency and moderate reliability of the scale measured (61) since the assessment consisted of 27 questions out of 46 having short scales (less than 5 items). In addition to that the overall questionnaire resulted from the combination of subsections having different value for Cronbach’s Alpha. In particular the set of questions related to the neophobia scale had α = 0.80, the lifestyle questions had α = 0.60, and food habits had α = 0.30. The general characteristics of both children participating in the study and the respondent caregivers are described in Table 1. The average age of the sample was 6.98 years (SD = 2.2) with the majority (70.7%) of the children aged 6–11 years. The questionnaire was completed primarily by mothers (88.8%) thus most of the respondents were aged 30–49 years (97%). The caregivers’ educational level qualification was balanced between upper secondary school (48.5%) and bachelor’s degree/master’s degree/Ph.D. (46.5%).

**TABLE 1:** General characteristics of children and parents/caregivers.

	Overall = 99
	*n* (%)
**Children**	
**Gender**	
Male	47 (47.5)
Female	52 (52.5)
**Age ranges**	
2–5 years	29 (29.3)
6–11 years	70 (70.7)
**Caregivers**	
**Type**	
Mother	88 (88.8)
Father	9 (9.1)
Others	2 (2.1)
**Age ranges**	
18–29 years	1 (1.0)
30–49 years	96 (97.0)
50–69 years	2 (2.0)
**Education**	
Lower secondary school	5 (5.0)
Upper secondary school	48 (48.5)
Bachelor’s degree/master’s degree/Ph.D	46 (46.5)

The average family size was 3.8 ± 0.66 individuals with more than half (62.6%) of the families consisting of four people, one-fourth of the families (25.3%) consisting of three individuals. Larger families were less common (11.1% five components and 1% seven people) (data not shown).

### Eating habits and lifestyle in the pre-pandemic period

#### Eating habits

Table 2 reports the eating habits in the pre-pandemic period of the assessed children. The dietary habits in line with the IDGs are those related to the consumption of whole grains (3–5 times a week for 58.6% of the sample), the prevalent use of olive oil as seasoning fat (95.9%) with limited use of butter or margarine (no serving for 83.9% of the sample). Behaviors that deviate far from the recommendations were the inadequate consumption of fruit (71.7%) and vegetables (58.6%), excessive consumption of red meat (>2 portions per week in 54.6% of the sample), inadequate consumption of fish (0–1 portion per week in 62.6% of the sample) and legumes (0–1 portion per week in 42.4% of the sample) and excessive consumption of sugary beverages with 19.2% of the sample that declare to consume them > 3 portions per week.

**TABLE 2:** Eating habits in the pre-pandemic period.

Food categories	Consumption frequencies *n* (%)			
*Vegetables/day* *1 serving* = *200 g*	***1***	***2***	**>*2***	***None***
	58 (58.6)	25 (25.3)	2 (2.0)	14 (14.1)
*Fruit/day* *1 serving* = *150 g*	***1–2***	***3***	**>*3***	***None***
	71 (71.7)	3 (3.1)	2 (2.0)	23 (23.2)
*White bread/day* *1 serving* = *50 g*	***1***	***2***	**>*2***	***None***
	54 (54.5)	27 (27.3)	9 (9.1)	9 (9.1)
*Whole-grain cereals (bread, pasta, rice*…*)/week*	***1–2***	***3–4***	**>*5***	***None***
	21 (21.2)	22 (22.2)	36 (36.4)	20 (20.2)
*Red meat, hamburgers, or meat products (ham, sausage, etc.)/week* *1 serving of meat* = *100 g* *1 serving of meat products* = *50 g*	***1***	***2***	**>*2***	***None***
	8 (8.1)	34 (34.3)	54 (54.6)	3 (3.0)

***What types of meat do you prefer to eat?***	***None***	***Mainly chicken and/or***	***All types of meat including beef,***	
		***turkey and/or rabbit***	***hamburger, pork, and lamb***	

	1 (1.0)	36 (36.4)	62 (62.6)	
*Fish/week* *1 serving* = *150 g*	***1***	***2***	**>*3***	***None***
	47 (47.5)	30 (30.3)	7 (7.1)	15 (15.1)
*Legumes/week* *1 serving of fresh legumes* = *150 g* *1 serving of soaked legumes* = *50 g*	***1***	***2***	**>*3***	***None***
	31 (31.3)	41 (41.4)	16 (16.2)	11 (11.1)
*Were the dishes consumed by the child seasoned exclusively with extra virgin olive oil?*	***Yes***		***No***	
	95 (95.9)		4 (4.1)	
*Butter and/or margarine/week* *1 serving* = *10 g*	***1***	***2***	**>*3***	***None***
	13 (13.1)	0 (0.0)	3 (3.0)	83 (83.9)
*Sugary beverages (orangeade, cola) including fruit juice/week*	***1***	***2***	**>*3***	***None***
	26 (26.3)	23 (23.2)	19 (19.2)	31 (31.3)
*Commercial sweets or pastries (not homemade) (cakes, cookies, sponge cake, or custard)/week*	***1***	***2***	**>*3***	***None***
	17 (17.2)	25 (25.2)	52 (52.5)	5 (5.1)
*Water/day*	**<1 L**	**1 L**	**1.5 L**	**>1.5 L**
	28 (28.3)	46 (46.5)	17 (17.1)	8 (8.1)

*n* and percentage values.

#### Lifestyle habits

Table 3 shows the lifestyle habits of the sample in the pre-pandemic situation. Catering service was present in 59.6% of the schools attended by the assessed children. However, not all the parents used the service, since the percentage of children who attended the school meal service was limited to 44.4%. Moreover, the survey showed that in the pre-pandemic period only 32.3% of the sample consumed all meals with the family. In the pre-pandemic period, physical activity levels, and the correspondent sedentary activities of the sample were very far from the recommendations, with 25.3% that declared not practicing physical activities and the rest of the sample that had a frequency of physical activity largely lower than the recommendations (55.5% 1–2 times/week and 16.2% 3–4 times/week). Among sedentary activities, it was found that a large part of the sample spent more time than recommended (1 h/day) in front of a screen (41.4% of the sample 2 h/day, 19.2% 3–4 h/day, and 3% more than 4 h per day). It is relevant to note that 40.4% of the sample used electronic devices during meals.

**TABLE 3:** Eating and lifestyles habits in the pre-pandemic period.

	Frequencies *n* (%)	
	
	Present	Absent
School catering	59 (59.6)	40 (40.4)
	**Used by parents**	**Not used by parents**
	44 (44.4)	55 (55.6)

**Family meals consumption**	**One** **meal**	**Two** **meals**	**Three** **meals**	**All meals**

	4 (4.1)	25 (25.2)	38 (38.4)	32 (32.3)
Screen time	**1 h**	**2 h**	**3–4 h**	**>4 h**
	36 (36.4)	41 (41.4)	19 (19.2)	3 (3.0)
Use of an electronic device during meals	**Yes**		**No**	
	40 (40.4)		59 (59.6)	

**Physical activity**	**Less frequently**	**1–2 times** **per** **week**	**3–4 times** **per** **week**	**>5** **times** **per** **week**	**Does not practice physical activity**

	1 (1.0)	55 (55.5)	16 (16.2)	2 (2.0)	25 (25.3)

Frequencies, *n*, and percentage values.

### Food neophobia in children

The child’s level of neophobia was measured using the CFNS. An average score of 37.8 (DS = 11.4) was obtained. No correlation was observed between the child’s age and neophobia (dependency ratio on average 0.03). As far as neophobia levels, the large majority of the sample (73.7%) showed an intermediate level of neophobia, 12.1% of the assessed children had a high level of neophobia, and 14.2% of the sample resulted in a low level of neophobia. Table 4 reports the association of neophobia levels with socio-demographic data and pre-pandemic food consumption of selected food groups. Based on the results, child gender (*p*-value 0.3) and parental education (*p*-value 0.7) are not associated with neophobia levels.

**TABLE 4:** The relationship between the level of neophobia and socio-demographic data, pre-pandemic food group consumption, *n* and percentage values.

		Level of neophobia *n* (%)		
		
		Low 14 (14.2)	Medium 73 (73.7)	High 12 (12.1)
**Socio-demographic data**				
Gender	Male	10 (19.2)	37 (71.2)	5 (9.6)
	Female	4 (8.5)	36 (76.6)	7 (14.9)
Parental education	Lower secondary school	0 (0)	5 (100.0)	0 (0.0)
	Upper secondary school	9 (18.8)	34 (70.8)	5 (10.4)
	Bachelor’s degree/master’s degree/Ph.D	5 (10.9)	34 (73.9)	7 (15.2)
Weight status	Underweight	0 (0.0)	6 (100.0)	0 (0.0)
	Normal weight	5 (9.8)	40 (78.4)	6 (11.8)
	Overweight	6 (27.3)	13 (59.1)	3 (13.6)
	Obese	3 (15.0)	14 (70.0)	3 (15.0)
**Pre-pandemic food groups consumption**				
Fruit consumption	No serving	2 (8.7)	17 (73.9)	4 (17.4)
	1–2	10 (14.1)	53 (74.6)	8 (11.3)
	3	1 (33.3)	2 (66.7)	0 (0.0)
	>3	1 (50.0)	1 (50.0)	0 (0.0)
Vegetable consumption	No serving	0 (0.0)	10 (71.4)	4 (28.6)
	1	9 (15.5)	41 (70.7)	8 (13.8)
	2	5 (20.0)	20 (80.0)	0 (0.0)
	>2	0 (0.0)	2 (100.0)	0 (0.0)
Legumes consumption	No serving	0 (0.0)	8 (72.7)	3 (27.3)
	1	4 (12.9)	24 (77.4)	3 (9.7)
	2	6 (14.6)	29 (70.7)	6 (14.6)
	>3	4 (25.0)	12 (75.0)	0 (0.0)
Whole grain cereals consumption	No serving	2 (10.0)	13 (65.0)	5 (25.0)
	1	5 (23.8)	16 (76.2)	0 (0.0)
	2	4 (18.2)	17 (77.3)	1 (4.5)
	≥5	3 (8.3)	27 (75.0)	6 (16.7)
Fish consumption	No serving	0 (0.0)	13 (86.7)	2 (13.3)
	1	4 (8.5)	35 (74.5)	8 (17.0)
	2	6 (20.0)	22 (73.3)	2 (6.7)
	>3	4 (57.1)*	3 (42.9)	0 (0.0)
Sugar beverages consumption	No serving	3 (9.7)	27 (87.1)	1 (3.2)
	1	2 (7.7)	22 (84.6)	2 (7.7)
	2	7 (30.4)*	11 (47.8)	5 (21.7)
	≥3	2 (10.5)	13 (68.4)	4 (21.1)

*p < 0.05. Calculated performing the Chi-square test with Bonferroni correction.

Neophobia-related food consumption was assessed in terms of the association between their pre-pandemic consumption and neophobia levels. The low consumption of fish was associated with neophobia (*p*-value < 0.05) in the sense that a high level of neophobia was observed in respondents that consumed limited quantities of fish. However, no statistically significant differences were observed between the level of neophobia and the consumption of vegetables (*p*-value 0.6), fruit (*p*-value 0.7), legumes (*p*-value 0.7), and whole-grain cereals (*p*-value 0.3). On the other hand, the highest consumption levels of sugary beverages were associated with higher levels of neophobia (*p*-value < 0.05).

Table 4 reports the children’s ponderal status, resulting in almost half (51%) of the respondents with normal weight, 22% with overweight, 20% with obesity, and 6% with underweight. Neophobia is not associated with ponderal status (*p*-value 0.5), however, in normal-weight children a particularly high prevalence of intermediate level of neophobia (78.4%) was found.

Weight status was compared with the child’s physical activity and parental education. In this sample, the risk of being overweight/obese is not related to the parents’ educational level (*p*-value 0.6) and to the physical activity of the child (*p*-value 0.9) (Supplementary Table 2).

### Eating habits and the family context during the second lockdown

As reported in Supplementary Table 3, worries caused by covid-19 pandemic did not influence the family’s eating habits for almost half of the sample (41.4%), however, a third of the respondents (34.3%) reported an influence of the concerns related to the pandemic on family eating habits. The lockdown largely impacted the habit of sharing meals which was reported by 32% of respondents (Table 3) before the pandemic and became 79% when affected by social restrictions (Table 5). Considering the long time spent at home, it was asked whether the child’s diet changed: in 43.4% of the cases no changes were reported; in 39.4% of the sample, the lockdown was an occasion to involve the children in the preparation of meals and, finally, in a minority of children (17.2%) the social isolation was characterized by moments of boredom compensated by excessive eating or with sedentary activities (Table 5). A significant correlation (*p*-value < 0.05) was found between the emotional consequences (stress, worries) of the pandemic and the changes in children’s eating habits. In particular, the majority (64.7%) of the children that experienced boredom, and consequently compensated with greater food intake, reported a family context that included worries about the COVID-19 pandemic (Table 5).

**TABLE 5:** Comparison between food groups’ consumption and changes in eating habits, conviviality during COVID-19 pandemic, *n* and percentage values.

			Changing eating habits			Sharing meals during the			
						COVID-19 pandemic			
				
Food groups	Food consumption	Total	No changing 43 (43.4)	Children have been involved in cooking 39 (39.4)	Using food as a reward 17 (17.2)	One meal 2 (2)	Two meals 4 (4)	Three meals 15 (15.2)	All meals 78 (78.8)
Pandemic vegetable consumption	Less	9 (9.1)	2 (22.2)	4 (44.4)	3 (33.3)	0 (0.0)	1 (11.1)	0 (0.0)	8 (88.9)
	Not changed	71 (71.7)	36 (50.7)	22 (31.0)	13 (18.3)	2 (2.8)	2 (2.8)	7 (9.9)	60 (84.5)
	More	19 (19.2)	5 (26.3)	13 (68.4)*	1 (5.3)	0 (0.0)	1 (5.3)	8 (42.1)*	10 (52.6)
Pandemic fruit consumption	Less	12 (12.1)	3 (25.0)	6 (50.0)	3 (25.0)	0 (0.0)	1 (8.3)	1 (8.3)	10 (83.4)
	Not changed	65 (65.6)	36 (55.4)	18 (27.7)	11 (16.9)	2 (3.1)	3 (4.6)	8 (12.3)	52 (80.0)
	More	22 (22.2)	4 (18.2)	15 (68.2)*	3 (13.6)	0 (0.0)	0 (0.0)	6 (27.3)	16 (72.7)
Pandemic legumes consumption	Less	4 (4.1)	0 (0.0)	0 (0.0)	4 (100.0)*	0 (0.0)	1 (25.0)*	0 (0.0)	3 (75.0)
	Not changed	74 (74.7)	37 (50.0)	26 (35.1)	11 (14.9)	1 (1.4)	2 (2.7)	8 (10.8)	63 (85.1)
	More	21 (21.2)	6 (28.6)	13 (61.9)	2 (9.5)	1 (4.8)	1 (4.8)	7 (33.3)*	12 (57.1)
Whole grain cereals consumption	Less	16 (16.2)	5 (31.2)	7 (43.8)	4 (25.0)	0 (0.0)	0 (0.0)	4 (25.0)	12 (75.0)
	Not changed	71 (71.7)	34 (47.9)	27 (38.0)	10 (14.1)	1 (1.4)	3 (4.2)	9 (12.7)	58 (81.7)
	More	12 (12.1)	4 (33.3)	5 (41.7)	3 (25.0)	1 (8.3)	1 (8.3)	2 (16.7)	8 (66.7)

**Effects of worries caused by**	**Degree of agreement**	**Total**	**No changing**	**Children have been involved**	**Using food to compensate**	**−**

Worries caused by pandemic changed eating habits	Completely disagree	20 (20.2)	16 (80.0)	2 (10.0)	2 (10.0)	**−**
	Disagree	21 (21.2)	9 (42.6)	9 (42.6)	3 (14.3)	
	Neither agree nor disagree	24 (24.2)	10 (41.6)	13 (54.2)	1 (4.2)	
	Agree	27 (27.3)	5 (18.5)	12 (44.5)	10 (37.0)	
	Completely agree	7 (7.1)	3 (42.8)	3 (42.8)	1 (14.3)	

*p < 0.05. Calculated performing the Chi-square test with Bonferroni correction.

Table 5 shows the results of questions related to the changing consumption of food groups normally associated with neophobia; the changing was qualitatively evaluated asking if the selected food consumption remained the same, increased or reduced. A large part of the sample reported no changes in the consumption of these foods. However, almost one-fifth of respondents reported an increase in the consumption of vegetables (19.2%), fruit (22.2%), legumes (21.2%), and whole-grain cereals (12.1%). The family context in terms of food habits during the second lockdown was found to be significantly associated with the changes in the consumption of fruit, vegetables and legumes (*p*-value < 0.05) in children’s diets. For more than half of the children that increased the consumption of fruit (68.2%), vegetables (68.4%), and legumes (61.9%) it was reported that lockdown was an opportunity to engage them in cooking activities as shown in Table 5. Involvement in kitchen was significantly associated with increased consumption of fruit and vegetables while the association did not reach the statistical significance for legume consumption. On the other hands reduced legume consumption was significantly associated with the behavior of using food to compensate for boredom. Family meal consumption (e.g., conviviality) was found to be significantly associated with vegetable and legume consumption during the second lockdown (*p*-value < 0.05). Combining the frequencies of the responses of sharing three or all meals with the family, as reported in the last two columns of Table 5, it is possible to see that the conviviality is particularly common in children who increased vegetable (94.7% the sum of 42.1 and 52.7%) and legume (90.4% the sum of 33.3 and 57.1%) consumption. On the other hand, fruit (*p*-value 0.7) and whole-grain cereal (*p*-value 0.6) consumption changes were not associated with family meal frequency during the second lockdown.

**FIGURE 1:**
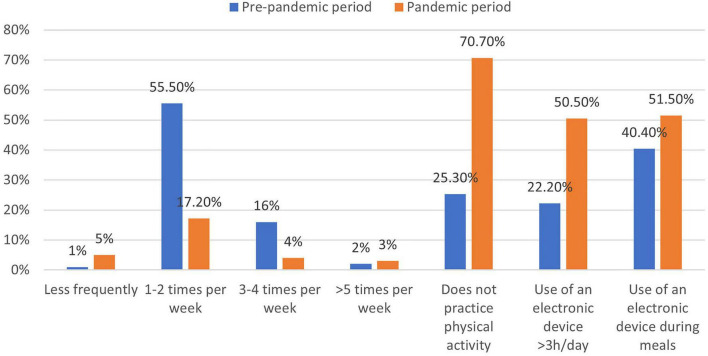
Comparisons of lifestyles between pre-pandemic and pandemic periods.

Figure 1 shows that lockdown largely impacted the level of physical activity. Compared with the pre-pandemic period, the percentage of children who do not practice physical activity increased to 70.7%, while among the remainder only a frequency of physical activity of 1–2 times a week was reported (17.2%); the highest frequencies of physical activity were uncommonly reported.

The sedentary attitude was characterized by a large screen-time behavior during the pandemic period in which the proportion of the sample spending 3–4 h in front of an electronic device passed from 19.2 to 36.4%, and the proportion of the children spending more than 4 h in front of a screen passed from 3 to 14.1%. Finally, compared to the pre-pandemic period, an increase in the use of screens during mealtime was observed (40.4% vs. 51.5%) (Figure 1).

**FIGURE 2:**
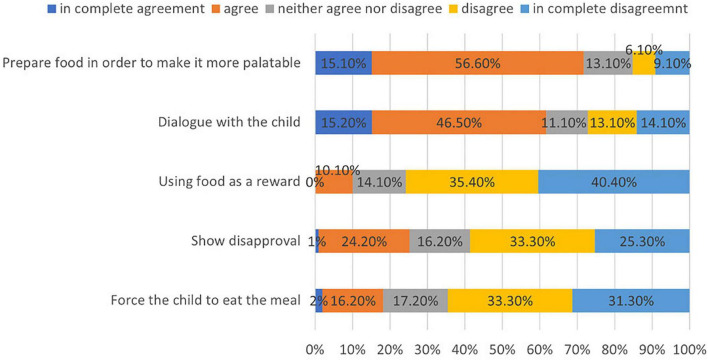
Educational strategies adopted in case of food refusal.

For almost the totality of children (96.9%) there was no worsening of food refusal during the second lockdown reported. Consistently, the parent in a large majority of cases (70.7%) did not experience difficulty in managing the refusals, which was claimed as a problem by 8.1% of the respondents. Figure 2 shows the results regarding the strategies adopted by parents when the child refused food. Most of them did not force the child to eat the meal (64.6%) (disagree/fully disagree), did not show disapproval (58.6%) (disagree/fully disagree), nor used the food as a reward (75.8%) (disagree/fully disagree). The most practiced feeding practices consisted of dialogue with the child (61.7%) (agree/fully agree) or increasing the palatability of the foods (71.7%) (agree/fully agree). No significant association was found between these items and the change in vegetable, fruit, whole-grain cereals, and legumes consumption in the second lockdown (*p*-value > 0.05) (Supplementary Tables 4–8).

Table 6 reports the association of neophobia levels with pandemic eating habits, lifestyles, and feeding practices. Parents showing disapproval toward food refusal is the only item that was found to be associated with neophobia (*p*-value < 0.05). Also, changes in the consumption of foods connected with neophobia during the pandemic period and the neophobia levels were not associated (*p*-value > 0.05). However, among children that increased the consumption of fruit, vegetables, legumes, and whole-grain cereals a higher percentage (93.1% vs. 84.3%) of children with intermediate/high level of neophobia was observed with respect to children that did not change their eating habits.

**TABLE 6:** The relationship between pandemic food groups’ consumption, use of the electronic device during meals, and educational strategies.

		Level of neophobia *n* (%)		
		
		Low 14 (14.2)	Medium 73 (73.7)	High 12 (12.1)
**Use of electronic device**				
Electronic devices during meals in pandemic period	Yes	8 (15.7)	34 (66.7)	9 (17.6)
	No	6 (12.5)	39 (81.2)	3 (6.3)
**Educational strategies**				
Pressure to eat	Completely disagree	8 (25.8)	19 (61.3)	4 (12.9)
	Disagree	5 (15.2)	24 (72.7)	4 (12.1)
	Neither agree nor disagree	0 (0)	15 (88.2)	2 (11.8)
	Agree	1 (6.2)	14 (87.5)	1 (6.2)
	Completely agree	0 (0.0)	1 (50.0)	1 (50.0)
Show disapproval	Completely disagree	8 (32.0)	11 (44.0)	6 (24.0)*
	Disagree	3 (9.1)	27 (81.8)	3 (9.1)
	Neither agree nor disagree	3 (18.8)	13 (81.2)	0 (0.0)
	Agree	0 (0.0)	22 (91.7)	2 (8.3)
	Completely agree	0 (0.0)	0 (0.0)	1 (100.0)
Using food as a reward	Completely disagree	10 (25.0)	27 (67.5)	3 (7.5)
	Disagree	2 (5.7)	27 (77.1)	6 (17.1)
	Neither agree nor disagree	1 (7.1)	11 (78.6)	2 (14.3)
	Agree	1 (10.0)	8 (80.0)	1 (10.0)
	Completely agree	0 (0.0)	0 (0.0)	0 (0.0)
Dialogue with the child	Completely disagree	2 (14.3)	10 (71.4)	2 (14.3)
	Disagree	1 (7.7)	11 (84.6)	1 (7.7)
	Neither agree nor disagree	2 (18.2)	6 (54.5)	3 (27.3)
	Agree	7 (15.2)	35 (76.1)	4 (8.7)
	Completely agree	2 (13.3)	11 (73.3)	2 (13.3)
Prepare food in order to make it more palatable	Completely disagree	2 (22.2)	5 (55.6)	2 (22.2)
	Disagree	0 (0.0)	6 (100.0)	0 (0.0)
	Neither agree nor disagree	2 (15.4)	8 (61.5)	3 (23.1)
	Agree	8 (14.3)	42 (75.0)	6 (10.7)
	Completely agree	2 (13.3)	12 (80.0)	1 (6.7)
**Pandemic food groups consumption**				
Pandemic fruit consumption	Greater	1 (4.5)	19 (86.4)	2 (9.1)
	Less	2 (16.7)	6 (50.0)	4 (33.3)
	Equal	11 (16.9)	48 (73.8)	6 (9.2)
Pandemic vegetables consumption	Greater	1 (5.3)	16 (84.2)	2 (10.5)
	Less	1 (11.1)	5 (55.6)	3 (33.3)
	Equal	12 (16.9)	52 (73.2)	7 (9.8)
Pandemic whole-grain cereals consumption	Greater	1 (8.3)	11 (91.7)	0 (0.0)
	Less	4 (25.0)	10 (62.5)	2 (12.5)
	Equal	9 (12.7)	52 (73.2)	10 (14.1)
Pandemic legumes consumption	Greater	2 (9.5)	18 (85.7)	1 (4.8)
	Less	0 (0.0)	3 (75.0)	1 (25.0)
	Equal	12 (16.2)	52 (70.3)	10 (13.5)

*p < 0.05. Calculated performing the Chi-square test with Bonferroni correction. *n* and percentage values.

As shown in Table 7, parents that did not experience difficulties in managing food refusal (disagree/fully disagree) tend to adopt conciliatory strategies to cope with this refusal, with 60.9% of cases that agreed and 73.3% fully agreed to have a dialogue with the child, 78.8 and 90.3% of cases that, respectively, disagreed and fully disagreed in forcing the child to eat the meal, in 69.7 and 96% of cases that, respectively, disagreed and fully disagreed in showing disapproval, and in 65.7% of cases that disagreed and 75% fully disagreed in using food as a reward (*p*-value < 0.05). On the other hand, the preparation of foods to increase palatability was not associated with parents’ difficulties in managing food refusal.

**TABLE 7:** The relationship between difficulty to manage the children’s food refusal and educational strategies.

		Difficulty to manage the refusal of food *n* (%)				
		
Educational strategies		Completely disagree 36 (36.4)	Disagree 34 (34.3)	Neither agree nor disagree 21 (21.2)	Agree 7 (7.1)	Completely agree 1 (1)
Pressure to eat	Completely disagree	24 (77.4)	4 (12.9)	2 (6.5)	1 (3.2)	0 (0.0)
	Disagree	7 (21.2)	19 (57.6)	7 (21.2)	0 (0.0)	0 (0.0)
	Neither agree nor disagree	2 (11.8)	7 (41.2)	6 (35.3)	2 (11.8)	0 (0.0)
	Agree	3 (18.7)	3 (18.7)	6 (37.5)	4 (25.0)	0 (0.0)
	Completely agree	0 (0.0)	1 (50.0)	0 (0.0)	1 (0.0)	1 (50.0)*
Show disapproval	Completely disagree	18 (72.0)*	6 (24.0)	0 (0.0)	1 (4.0)	0 (0.0)
	Disagree	9 (27.3)	14 (42.4)	9 (27.3)	1 (3.0)	0 (0.0)
	Neither agree nor disagree	3 (18.7)	8 (50.0)	4 (25.0)	0 (0.0)	1 (6.2)
	Agree	6 (25.0)	6 (25.0)	7 (29.2)	5 (20.8)	0 (0.0)
	Completely agree	0 (0.0)	0 (0.0)	1 (100.0)	0 (0.0)	0 (0.0)
Using food as a reward	Completely disagree	24 (60.0)*	6 (15.0)	8 (20.0)	1 (2.5)	1 (2.5)
	Disagree	4 (11.4)	19 (54.3)	7 (20.0)	5 (14.3)	0 (0.0)
	Neither agree nor disagree	4 (28.6)	6 (42.8)	4 (28.6)	0 (0.0)	0 (0.0)
	Agree	4 (40.0)	3 (30.0)	2 (20.0)	1 (10.0)	0 (0.0)
	Completely agree	0 (0.0)	0 (0.0)	0 (0.0)	0 (0.0)	0 (0.0)
Dialogue with the child	Completely disagree	10 (71.4)	2 (14.3)	2 (14.3)	0 (0.0)	0 (0.0)
	Disagree	5 (38.4)	4 (30.8)	3 (23.1)	1 (7.7)	0 (0.0)
	Neither agree nor disagree	8 (72.7)	2 (18.2)	1 (9.1)	0 (0.0)	0 (0.0)
	Agree	8 (17.4)	20 (43.5)	13 (28.3)	5 (10.8)	0 (0.0)
	Completely agree	5 (33.3)	6 (40.0)	2 (13.3)	1 (6.7)	1 (6.7)*
Prepare food in order to make it more palatable	Completely disagree	6 (66.7)	2 (22.2)	1 (11.1)	0 (0.0)	0 (0.0)
	Disagree	2 (33.3)	3 (50.0)	1 (16.7)	0 (0.0)	0 (0.0)
	Neither agree nor disagree	5 (38.5)	4 (30.7)	4 (30.7)	0 (0.0)	0 (0.0)
	Agree	18 (32.1)	21 (37.5)	12 (21.4)	5 (8.9)	(0.0)
	Completely agree	5 (33.3)	4 (26.7)	3 (20.0)	2 (13.3)	1 (6.7)

*p < 0.05. Calculated performing the Chi-square test with Bonferroni correction. *n* and percentage values.

## Discussion

The objective of this experimental work was to evaluate the behavioral changes that occurred during the COVID-19 pandemic period and the relation between eating habits and parental attitude toward neophobia in a sample of children living in Lazio, an Italian central region. Food neophobia is a very common behavior among children, without distinction of gender, especially in the period from 2 to 6 years. In this developmental period children acquire more autonomy, becoming more neophobic; the neophobic attitude tends to progressively reduce during adulthood (3). The results of the present study confirm the extent of the problem, since more than half of the sample (73.7%) has an intermediate level of neophobia and 12.1% a high level, still without distinction between males and females. Food neophobia in children has also been assessed in other Italian studies (23, 62). Although different prevalence emerged [26.5% low, 44.3% medium, and 29.2% high level of neophobia (23); 24% low, 53.8% medium, and 22.1% high level of neophobia (62)], it should be pointed out that in the other studies the intermediate level of neophobia was consistent with the results of the present study. However, considering that the different Authors used different neophobia scales, comparisons must be made with caution.

Childhood obesity in Italy, as in other industrialized European countries, represents a priority public health problem. A surveillance system on overweight and obesity and related risk factors has been activated in Italian primary schools (45) which began in 2008. The latest survey (63) reports that in the Lazio region the prevalence of ponderal excess accounted for 30.8%, this means that our sample showed a particularly high prevalence of overweight and obesity (42%) in comparison with the rest of the region. This finding is probably related to the fact that our sample from the south of the Lazio region bordering the Italian region with the highest prevalence of children overweight (Campania region, 44.2%) (64). It has been hypothesized that food neophobia may contribute to childhood obesity, because to compensate for the child’s rejection of food, parents offer them a more palatable and acceptable alternative, such as high-calorie foods rather than healthy foods such as fruits and vegetables, consequently increasing of the risk of excess weight. Our study confirms the absence of association between neophobia and ponderal status which was also reported in several other papers (19, 21, 23). Deserving comment, however, is the observation that a high prevalence of children with an intermediate level of neophobia (78.4%) are among those with normal weight. These results are consistent with the literature that defines food neophobia as a natural stage of child development that does not impair growth rate (65). We could speculate that the ambivalence of the results of the studies comparing weight status and food neophobia depends on the parental feeding style. In the case of parents that counteract neophobia with dialogue and a non-constrictive approach, the child would probably maintain a normal weight; otherwise, if neophobia is addressed by offering palatable and calory-dense foods to compensate for the non-acceptance of healthy foods, the child might develop overweight or obesity.

The consumption of foods most commonly associated with food neophobia was evaluated in the studied sample. The results showed low consumption of foods typical of the Mediterranean model (66) such as fruit, vegetables, and legumes, and, on the contrary, high consumption of foods typical of a Western dietary model (67), such as sweets, sugary beverages, and red meat. Therefore, the results of this study support the literature that reports a trend toward the abandonment of the MD with a shift toward a more Westernized dietary patterns in children living in industrialized countries (1). Several behavioral factors may be the cause of this phenomenon including the habits of purchasing ready meals or foods that are easy to prepare as a consequence of limited time to prepare fresh foods (1). However, neophobia could also be one among many factors contributing to the mentioned shift in food consumption patterns. Neophobia typically occurs with highly recommended foods such as fruits, vegetables, and legumes, which have a bitter or acidic taste, it also typically occurs with animal source food, such as fish (68). The results of the present study showed that the consumption of these foods, although not in line with the recommendations for a large part of the sample, was not associated with the level of neophobia, except for the consumption of fish, which was less consumed by neophobic children. Other studies in the literature document poor adherence to nutritional recommendations in children with neophobia (17); actually in the present assessment we could confirm that food neophobia limits dietary variety and quality.

The pandemic impacted eating habits as shown in different studies conducted in Italy during lockdown, showing that the consumption of fruits, vegetables, and legumes did not change (28) or in some cases improved (32, 34). However, other studies showed an increase in the consumption of sweet or salty snacks with high energy density, sugar beverages, and red meat (30, 34). In this study, for the majority of the sample, eating habits did not change and, notably, an increased consumption of fruits, vegetables, and legumes was observed in approximately 20% of children. The fact that the pandemic period also impacted some eating habits positively was confirmed in other studies (47, 69).

A relevant aspect that emerges from this study concerns the identification of factors that could be responsible for the variation of children’s eating habits during the lockdown. Van der Horst (39) reported that the involvement of children in the preparation of meals contributes to improving the quality of the diet and their consumption of vegetables (39), as it represents a strategy to help children become familiar with the non-accepted foods. Consistently, in the present study, for more than half of the children that increased the consumption of fruit, vegetables, and even with not significant association, of legumes it was reported that lockdown was an opportunity to engage them in cooking activities. These data confirm that involving children in meal preparation is an effective strategy for increasing vegetable consumption and reducing food neophobia (70).

During the pandemic period, due to social isolation, it was reported an increase in the number of meals consumed in the family (32) and this is confirmed by the results of this study showing that the percentage of children who consumed all meals in the presence of their parents increased from 32.3 to 78.8%. In addition, family meal consumption was found to be significantly associated with the consumption of vegetables and legumes during the lockdown: almost all children in whom there was increased consumption of vegetables (94.7%) and legumes (90.4%) had shared three or all meals with the family. We could interpret these data considering the findings of Lumeng et al. (71) which reported that the eating habits adopted in the family influenced children’s food choices because, through a process of observation and imitation called modeling (72), the child learns to accept new foods. In this study, the healthy eating habits of parents and increased family mealtimes contributed to influencing the eating habits of the children as described by Litterbach and co-workers (73).

The use of electronic devices during mealtime is associated with increased energy intake and risk of childhood obesity, but also with the risk of worsening the level of food neophobia as the presence of distractors during mealtime (e.g., TV) has been shown to lead children to refuse more food (74). In this study, screen time was observed to be significantly increased during the lockdown, especially the use of these devices during the meal (51.5% vs. 40.4%) as shown in Figure 1, however, there was not a significant association found between screen time and the child’s level of food neophobia (Table 6).

The feeding practice most adopted by the parents in the studied sample were dialogue and the preparation of the not preferred foods in a more palatable way. The only strategy associated with the level of neophobia is the parents’ disapproval. The feeding practices adopted by parents at mealtime may also influence the child’s eating habits and level of neophobia. Forcing the child to consume the proposed food, showing disapproval (75–78), or using food as a reward (79) are considered strategies with limited impact, perhaps even worsening the children’s attitude. Actually in the studied sample it was observed a reduction in legume consumption in children with parents that used foods to compensate for boredom probably because the typology of foods used as a reward is more likely to be products nearest to the children requests, such as salty or sweet items. In contrast, an open attitude based on dialogue with convincing themes (80) or preparing the food to make it more palatable (81) are considered most efficient.

Data on neophobia during the pandemic period were scarce; in a study conducted on Brazilian children during the pandemic period, it was hypothesized that children’s eating habits and behaviors were affected by the pandemic with a consequent increase in the level of neophobia (82). This hypothesis is not confirmed by our data which demonstrated that the family context influences the eating habits and the eating behavior of the child. In the present study, the families particularly worried about the pandemic adopted food consumption behaviors aimed to counteract the increased boredom, embracing eating as a compensatory strategy. However, no greater rejection of selected food by the child was observed and therefore no worsening of the level of neophobia.

The majority of parents (70.7%) did not experience difficulties in managing the children’s refusal of food. In particular, the correlations between the parent’s ability to manage this refusal and the feeding practices adopted showed that in the absence of difficulties, in most cases the parent preferred dialogue. Parents also demonstrated disagreement with the adoption of forcing strategies such as pressuring the child to eat, disapproval or using food as a reward.

Outside the family context and parents’ behaviors, different socioenvironmental factors may contribute to the development of food neophobia. In a study carried out in school age children in Saudi Arabia a significant positive association between peer modeling and cognitive factors and the occurrence of food neophobia was found (78). Early taste experience, prenatal food exposure and breastfeeding and complementary feeding habits, are associated with food choices later in life (83). These periods are largely influenced by parents’ attitude, however, according to Ventura and Worobey (84) social influences become increasingly important for the development of food preferences throughout infancy, and may either support or contrast the preferences learned during the prenatal period. Moreover, the early postnatal periods and the factors that influence the food habit changes that occur become more complex through the years. Particularly relevant is the analysis of the relationship between food neophobia occurrence in the vulnerable population groups. Low-income Brazilian preschoolers with a high level of food neophobia showed a lower adherence to traditional dietary patterns and distinct food preferences than their peers with low-middle food neophobia. Therefore, neophobic Brazilian children were more likely to eat ultra-processed foods, such as chips, cookies, and sweets (85). The retrospective cross-sectional design that we applied as well as our sample size did not permit the differentiation between the influence of other variables such as income and educational level as confounding factors on the occurrence of food neophobia. However, in our sample, the neophobic children showed a low consumption of foods typical of the Mediterranean model such as fruit, vegetables, and legumes confirming the parallelism between neophobia and low adherence to traditional dietary pattern as reported in the study of Anjos et al. (85).

This study has strengths and limitations. The main strength is the fact that the study provided a picture of a very particular moment in which the daily life of people and in particular of children were largely affected either in a positive or negative sense and any data and findings that contributed to explaining these moments are important to be described and shared. In terms of limitations, first, the study design involved the administration of an online self-completed questionnaire, with limited possibility to verify if the response corresponds to a real attitude and behaviors or was a reaction to a question influenced by accepted social norms. The evaluation of the bias of a self-administrated questionnaire compared to an evaluation mediated by an interviewer is complex and not univocal, in the sense that the presence of an interviewer is not always an advantage in terms of control of the quality of response. Large numbers of studies, especially during pandemic period, rely on self-reported information and the validity of self-reported data is an aspect to be discussed. Self-reported data are accurate when individuals understand the questions and when there is a strong sense of anonymity and little fear of reprisal, all aspects that increase the validity of the results (86). However, no survey is perfect, and there is always a certain margin of error. The issue of the anonymity is further mentioned by Althubaiti (87) stating that self-reporting data can be affected by an external bias caused by social desirability or approval, especially in cases where anonymity cannot be guaranteed at the time of data collection. Actually with the online system and the confidentiality that we established, which necessary in consideration of the Italian and European normative framework, these aspects were largely assured. A further limitation of the study was the fact that the questionnaire was constructed to carry out a retrospective survey, therefore, the reliability of the answers depends on the memory of the respondents. However, the recall period was relatively short and we asked for routine and usual habits that are reported as elements to minimize the recall bias (87). An important limitation was the small sample size; in this study the sample size was small because the pandemic limited the possibility to reach a large number of participants. In consideration of the target (children) it would have been useful to establish a direct contact with the respondent families that was not possible for effect of the social constraints of the lockdown. With the present sample size, our study has a precision level of 9%. There is not an accepted guideline for choosing an appropriate precision level, some authors recommended selecting a precision level of 5% if the prevalence of the main outcome is going to be between 10 and 90% (60). In this study we used the prevalence of 26% of food neophobia (16) hence our precision level could be considered low. However according to Button et al. (88) the median statistical power of studies in the neurosciences, in which neophobia is included, is approximately between 8 and 31%, hence in our case the precision level is in line with other similar studies. The cross-sectional retrospective design with the same subjects that were assessed in pre and post pandemic conditions minimized the effect of confounding factors. However this self-controlled design has a major limitation the applicability that is circumscribed to a narrow set of situations (89). For all these reasons, as prudential attitude, we avoided too much conclusive considerations and generalization of the outcomes of this study, limiting the observations in the poll of people living in the very defined territory (south province of an Italian central region). Nonetheless it would be advisable to extend the study to a larger number of individuals, more evenly distributed throughout the country, to give greater solidity to the conclusions drawn.

## Conclusion

In conclusion, in the sample of children analyzed in the present work, the level of neophobia was not affected by the pandemic period and globally, the assessed children did not experience an increase in selected food refusal. Consistently, parents did not perceive difficulties in managing their child’s refusal of food, and therefore feeding practices were not coercive, but based on dialogue or using the expedient of preparation of the most palatable foods.

For most of the assessed children, eating habits did not change compared with the pre-pandemic period with a subset of them that improved their eating habits based on the effect of a larger involvement in food preparation and greater frequency of family meals. This study, even with the limited sample size, confirms the effectiveness of these strategies as tools to increase vegetable consumption and mitigate food neophobia. It also suggests that the pandemic, and especially social isolation, in this group of children could positively affect food neophobia if it was an opportunity to share more family meals or if the long time spent at home was capitalized on to involve children in food preparation, helping them to become familiar with new foods.

## Data availability statement

The raw data supporting the conclusions of this article will be made available by the authors, without undue reservation.

## Author contributions

ADN and US carried out the research questions, conceptualization, and design of the study. LR and FG revised the methodology. ADN and FG carried out the database compilation and data analysis. ADN carried out the manuscript writing and original draft preparation. LR, FG, and US did the writing, review, and editing. All authors have read and agreed to the published version of the manuscript.
