# Supplementary material for: The impact of COVID-19 pandemic on food habits and neophobia in children in the framework of the family context and parents’ behaviors: A study in an Italian central region
Source: Front Nutr. 2022 Dec 8;9:1070388. doi: 10.3389/fnut.2022.1070388 (PMC9772991; doi:10.3389/fnut.2022.1070388)
Supplement: Supplementary file 1 [file Data_Sheet_1.docx]

Supplementary Material

***Table S1: The Questionnaire***

| **Questions** | | **Answers** |
| --- | --- | --- |
| **SOCIO-DEMOGRAPHIC DATA** | | |
| **Who filled out the questionnaire?** | | **Mother/Father/Others** |
| **Age (years)** | | 18-29/30-49/50-69/70-79/>80 |
| **Level of education** | | Primary school/Middle school/High school/Bachelor’s degree/ Master’s degree/PhD |
| **How many people live with you?** | | People number |
| **Kids less than 11 are present in the family?** | | Yes/No |
| **Age of child** | | Age in years |
| **Gender** | | Male/Female |
| **Weight** | | kg |
| **Height** | | cm |
| **SECTION 1- Eating habits and lifestyle of the child in the pre-pandemic period** | | |
| **How many servings of fruit did the child eat per day? 1 serving= 150 g** | | None/1-2/3/>3 |
| **How many servings of vegetables did the child eat per day? 1 serving = 200 g** | | None/1/2/>2 |
| **How many servings of white bread did the child eat per day? 1 serving = 50** | | None/1/2/>2 |
| **How many times did the child eat whole grain cereals (e.g. pasta, rice, bread) per week?** | | None/1-2/3-4 />4 |
| **How many servings of red meat, hamburger, or processed meat (e.g., cured ham, salami) did the child eat per week? 1 serving of red meat = 100 g; 1 serving of processed meat = 50 g** | | None/1/2/>2 |
| **Which kinds of meat did the child prefer eating?** | | Mainly white meat (chicken, turkey, or rabbit)/Every type of meat, including red meat (beef, pork, and lamb)/None |
| **How many servings of fish or shellfish did the child eat per week? 1 fish serving = 150** | | None/1/2/>3 |
| **How many servings of legumes did the child eat per week? 1 serving of fresh legumes = 150 g; 1 serving of dry legumes = 50 g** | | None/1/2/>3 |
| **Were the dishes consumed by the child seasoned exclusively with extra virgin olive oil?** | | Yes/No |
| **How many servings of butter and/or margarine did the child eat per week? 1 serving = 10 g** | | None/1/2/>3 |
| **How many sugary drinks (e.g., coke, orange soda), including fruit juice, did the child drink per week?** | | None/1/2/>3 |
| **How many times did the child eat sweets or pastries (e.g., biscuits, croissants, cakes, or custard) per week?** | | None/1/2/>3 |
| **How much water did the child drink daily?** | | Less than 1 l/Up to 1 l/Up to 1,5 l/More than 1,5 l |
| **Is the school attended by the child provided with food service?** | | Yes/No |
| **Was the child consuming lunch at the food service?** | | Yes/No |
| **In the pre-pandemic period what meals were consumed by family?** | | Choose 1 or more of the following options: Breakfast/snack/lunch/dinner |
| **Did the child engage in physical activity?** | | Yes/No |
| **How many times a week did the child engage in physical activity?** | | He/She did not do physical activity/less frequently/1-2 times per week/3-4 times per week/5 and more times per week |
| **How much time did the child spend in front of a media device (e.g. pc, tv, tablet)?** | | 1h/2h/3-4h/>4h |
| **Was the child looking at a video screen during the meal?** | | Yes/No |
| **SECTION 2- Eating habits and lifestyle of the child during the second lockdown** | | |
| **Have the concerns caused by the pandemic affected family eating habits?** | Choose one of the following options: Strongly disagree/Disagree/Neither agree nor disagree/Agree/Strongly agree | |
| **During the second lockdown what meals were consumed with family?** | Choose 1 or more of the following options: Breakfast/snack/lunch/dinner | |
| **How have the child's eating habits changed?** | | His/Her eating habits have not changed/ He/She has been involved in cooking and he/her is learning new things about nutrition/ He/She was bored and for this reason, food and sedentary activities are compensatory |
| **Did the child show greater rejection of food?** | | Yes/No |
| **Parental educational strategies**  Did you find it more difficult to manage the child's refusal of food?  Did you force the child to eat the meal?  Did you show disapproval if the child did not eat?  Did you encourage the child to eat using food as a reward (e.g. "I'll buy you ice cream/toy if you finish eating")?  Did you talk to the child to persuade him to eat (e.g. commenting on the food to be consumed)?  Have you prepared the food to make it more palatable to the child (e.g. meatballs, flans)? | | For each question, choose one of the following options:  Strongly disagree/Disagree/Neither agree nor disagree/Agree/Strongly agree |
| **During the second lockdown, how was the consumption of the following food categories? Increased, decreased, or the same as before**  Fruit/Vegetables/ Whole grain cereals /legumes | | |
| **Did the child engage in physical activity during the second lockdown?** | | Yes/No |
| **How many times a week did the child engage in physical activity?** | | He/She did not do physical activity/less frequently/1-2 times per week/3-4 times per week/5 and more times per week |
| **How much time did the child spend in front of a media device (e.g. pc, tv, tablet)?** | | 1h/2h/3-4h/>4h |
| **Was the child looking at a video screen during the meal?** | | Yes/No |
| **SECTION 3 – Evaluation of the child’s neophobic behavior** | | |
| The child constantly samples new and different food  The child doesn't trust new foods  If the child does not know what a kind of food is, he does not try it  The child likes food from different cultures  Ethnic food looks to him/her too weird to eat  During a party, the child is willing to try new foods  The child is afraid to eat things he has never had before  The child is very particular about the food he/she eats  The child will eat almost anything  The child likes to try new ethnic restaurants | | For each question, choose one of the following options:  Strongly disagree/Disagree/Somewhat disagree/Neither agree nor disagree/Somewhat agree/Agree/Strongly agree |

***Table S2: Comparison between weight status with parental education and level of physical activity***

|  | **Weight status** | | | | | |
| --- | --- | --- | --- | --- | --- | --- |
|  | **Underweight** | | **Normal weight** | **Overweight** | | **Obesity** |
| **Parental education** |  | |  |  | |  |
| **Lower secondary schools** | 1 (20.0%) | | 1 (20.0%) | 1 (20.0%) | | 2 (40.0%) |
| **Upper secondary schools** | 2 (4.2%) | | 27 (56.2%) | 10 (20.8%) | | 9 (18.8%) |
| **Bachelor’s degree/Master’s degree/PhD** | 3 (6.5%) | | 23 (50.0%) | 11 (23.9%) | | 9 (19.6%) |
| **Level of physical activity** |  | | | | | |
| **Not practice physical activity** | 2 (8.0%) | 13 (52.0%) | | 4 (16.0%) | 6 (24.0%) | |
| **Less frequently** | 0 (0.0%) | 1 (100.0%) | | 0 (0.0%) | 0 (0.0%) | |
| **1-2 times per week** | 3 (5.5%) | 31 (56.4%) | | 13 (23.6%) | 8 (14.5%) | |
| **3-4 times per week** | 1 (6.3%) | 5 (31.2%) | | 4 (25.0%) | 6 (37.5%) | |
| **≥ 5 times per week** | 0 (0.0%) | 1 (50.0%) | | 1 (50.0%) | 0 (0.0%) | |

***Table S3: Influence of worries caused by the pandemic on eating habits***

|  | **Overall = 99**  **n (%)** |
| --- | --- |
| **Have the concerns caused by the pandemic affected eating habits?** |  |
| **Completely disagree** | 21 (21.2%) |
| **Disagree** | 20 (20.2%) |
| **Neither agree nor disagree** | 24 (24.2%) |
| **Agree** | 27 (27.3%) |
| **Completely agree** | 7 (7.1%) |

***Table S4: Comparison between eating habits during the second lockdown and pressure to eat.***

| **Educational strategy** | **Food groups** | | | | | | | | | | | |
| --- | --- | --- | --- | --- | --- | --- | --- | --- | --- | --- | --- | --- |
| **Pressure to eat** | **Pandemic fruit consumption** | | | **Pandemic vegetable consumption** | | | **Pandemic legumes consumption** | | | **Pandemic whole-grain consumption** | | |
|  | **Less**  12 (12.1%) | **Not change**  65 (65.6%) | **More**  22 (22.2%) | **Less**  9 (9.1%) | **Not change**  71 (71.7%) | **More**  19 (19.2%) | **Less**  4 (4.1%) | **Not change**  74 (74.7%) | **More**  21 (21.2%) | **Less**  16 (16.2%) | **Not change**  71 (71.7%) | **More**  12  (12.1%) |
| **Completely disagree** | 5 (41.7%) | 21 (32.3%) | 5 (22.7%) | 5 (55.5%) | 24 (33.8%) | 2 (10.5%) | 1 (25-0%) | 28 (37.8%) | 2 (9.5%) | 6 (37.5%) | 25 (35.2%) | 0 (0.0%) |
| **Disagree** | 2 (16.7%) | 19 (29.2%) | 12 (54.5%) | 0 (0.0%) | 25 (35.2%) | 8 (42.1%) | 1 (25.0%) | 23 (31.1%) | 9 (42.8%) | 6 37.5%) | 20 (28.2%) | 7 (58.3%) |
| **Neither agree nor disagree** | 2 (16.7%) | 11 (16.9%) | 4 (18.2%) | 3 (33.3%) | 9 (12.7%) | 5 (26.3%) | 2 (50.0%) | 10 (13.5%) | 5 (23.8%) | 3 (18.7%) | 11 (15.5%) | 3 (25.0%) |
| **Agree** | 2 (16.7%) | 13 (20%) | 1 (4.5%) | 1 (11.1%) | 11 (15.5%) | 4 (21.1%) | 0 (0.0%) | 12 (16.2%) | 4 (19.0%) | 1 (6.2%) | 13 (18.3%) | 2 (16.7%) |
| **Completely agree** | 1 (8.3%) | 1 (1.5%) | 0 (0.0%) | 0 (0.0%) | 2 (2.8%) | 0 (0.0%) | 0 (0.0%) | 1 (1.4%) | 1 (4.8%) | 0 (0.0%) | 2 (2.8%) | 0 (0.0%) |

***Table S5: Comparison between eating habits during the second lockdown and showing disapproval.***

| **Educational strategy** | **Food groups** | | | | | | | | | | | |
| --- | --- | --- | --- | --- | --- | --- | --- | --- | --- | --- | --- | --- |
| **Showing disapproval** | **Pandemic fruit consumption** | | | **Pandemic vegetable consumption** | | | **Pandemic legumes consumption** | | | **Pandemic whole-grain consumption** | | |
|  | **Less**  12 (12.1%) | **Not change**  65 (65.6%) | **More**  22 (22.2%) | **Less**  9 (9.1%) | **Not change**  71 (71.7%) | **More**  19 (19.2%) | **Less**  4 (4.1%) | **Not change**  74 (74.7%) | **More**  21 (21.2%) | **Less**  16 (16.2%) | **Not change**  71 (71.7%) | **More**  12  (12.1%) |
| **Completely disagree** | 5 (41.7%) | 17 (26.2%) | 3 (13.6%) | 5 (55.5%) | 18 (25.3%) | 2 (10.5%) | 1 (25.0%) | 22 (29.7%) | 2 (9.5%) | 3 (18.7%) | 21 (29.6%) | 1 (8.3%) |
| **Disagree** | 2 (16.7%) | 19 (29.2%) | 12 (54.5%) | 1 (11.1%) | 23 (32.4%) | 9 (47.4%) | 2 (50.0%) | 21 (28.4%) | 10 (47.6%) | 7 (43.7%) | 21 (29.6%) | 5 (41.7%) |
| **Neither agree nor disagree** | 3 (25.0%) | 11 (16.9%) | 2 (9.1%) | 1 (11.1%) | 13 (18.3%) | 2 (10.5%) | 1 (25.0%) | 13 (17.6%) | 2 (9.5%) | 2 (12.5%) | 11 (15.5%) | 3 (25.0%) |
| **Agree** | 2 (16.7%) | 17 (26.2%) | 5 (22.7%) | 2 (22.2%) | 16 (22.6%) | 6 (31.6%) | 0 (0.0%) | 17 (22.9%) | 7 (33.4%) | 4 (25.0%) | 17 (23.9%) | 3 (25.0%) |
| **Completely agree** | 0 (0.0%) | 1 (1.5%) | 0 (0.0%) | 0 (0.0%) | 1 (1.4%) | 0 (0.0%) | 0 (0.0%) | 1 (1.4%) | 0 (0.0%) | 0 (0.0%) | 1 (1.4%) | 0 (0.0%) |

***Table S6: Comparison between eating habits during the second lockdown and dialogue.***

| **Educational strategy** | **Food groups** | | | | | | | | | | | |
| --- | --- | --- | --- | --- | --- | --- | --- | --- | --- | --- | --- | --- |
| **Dialogue** | **Pandemic fruit consumption** | | | **Pandemic vegetable consumption** | | | **Pandemic legumes consumption** | | | **Pandemic whole-grain consumption** | | |
|  | **Less**  12 (12.1%) | **Not change**  65 (65.6%) | **More**  22 (22.2%) | **Less**  9 (9.1%) | **Not change**  71 (71.7%) | **More**  19 (19.2%) | **Less**  4 (4.1%) | **Not change**  74 (74.7%) | **More**  21 (21.2%) | **Less**  16 (16.2%) | **Not change**  71 (71.7%) | **More**  12  (12.1%) |
| **Completely disagree** | 2 (16.7%) | 11 (16.9%) | 1 (4.5%) | 3 (33.3%) | 11 (15.5%) | 0 (0.0%) | 2 (50.0%) | 12 (16.2%) | 0 (0.0%) | 2 (12.5%) | 12 (16.9%) | 0 (0.0%) |
| **Disagree** | 0 (0.0%) | 8 (12.3%) | 5 (22.7%) | 0 (0.0%) | 12 (16.9%) | 1 (5.3%) | 1 (25.0%) | 9 (12.2%) | 3 (14.3%) | 1 (6.3%) | 9 (12.7%) | 3 (25.0%) |
| **Neither agree nor disagree** | 0 (0.0%) | 8 (12.3%) | 3 (13.6%) | 0 (0.0%) | 7 (9.9%) | 4 (21.0%) | 0 (0.0%) | 10 (13.5%) | 1 (4.8%) | 4 (25.0%) | 7 (9.8%) | 0 (0.0%) |
| **Agree** | 7 (58.3%) | 29 (44.6%) | 10 (45.5%) | 4 (44.4%) | 30 (42.2%) | 12 (63.2%) | 0 (0.0%) | 34 (45.9%) | 12 (57.1%) | 7 (43.7%) | 32 (45.1%) | 7 (58.3%) |
| **Completely agree** | 3 (25.0%) | 9 (13.8%) | 3 (13.6%) | 2 (22.2%) | 11 (15.5%) | 2 (10.5%) | 1 (25.0%) | 9 (12.2%) | 5 (23.8%) | 2 (12.5%) | 11 (15.5%) | 2 (16.7%) |

***Table S7: Comparison between eating habits during the second lockdown and preparing food to make it more palatable.***

| **Educational strategy** | **Food groups** | | | | | | | | | | | |
| --- | --- | --- | --- | --- | --- | --- | --- | --- | --- | --- | --- | --- |
| **Prepare food to make it more palatable** | **Pandemic fruit consumption** | | | **Pandemic vegetable consumption** | | | **Pandemic legumes consumption** | | | **Pandemic whole-grain consumption** | | |
|  | **Less**  12 (12.1%) | **Not change**  65 (65.6%) | **More**  22 (22.2%) | **Less**  9 (9.1%) | **Not change**  71 (71.7%) | **More**  19 (19.2%) | **Less**  4 (4.1%) | **Not change**  74 (74.7%) | **More**  21 (21.2%) | **Less**  16 (16.2%) | **Not change**  71 (71.7%) | **More**  12  (12.1%) |
| **Completely disagree** | 1 (8.3%) | 7 (10.8%) | 1 (4.5%) | 2 (22.2%) | 6 (8.5%) | 1 (5.3%) | 2 (50.0%) | 7 (9.5%) | 0 (0.0%) | 3 (18.9%) | 6 (8.5%) | 0 (0.0%) |
| **Disagree** | 0 (0.0%) | 6 (9.2%) | 0 (0.0%) | 0 (0.0%) | 5 (7.0%) | 1 (5.3%) | 0 (0.0%) | 6 (8.1%) | 0 (0.0%) | 0 (0.0%) | 5 (7.0%) | 1 (8.3%) |
| **Neither agree nor disagree** | 2 (16.7%) | 9 (13.8%) | 2 (9.1%) | 3 (33.3%) | 9 (12.7%) | 1 (5.3%) | 0 (0.0%) | 12 (16.2%) | 1 (4.8%) | 0 (0.0%) | 11 (15.5%) | 2 (16.7%) |
| **Agree** | 8 (66.7%) | 33 (50.8%) | 15 (68.2%) | 4 (44.4%) | 40 (56.3%) | 12 (63.1%) | 2 (50.0%) | 37 (50.0%) | 17 80.9%) | 9 (56.2%) | 39 (54.9%) | 8 (66.7%) |
| **Completely agree** | 1 (8.3%) | 10 (15.4%) | 4 (18.2%) | 0 (0.0%) | 11 (15.5%) | 4 (21.0%) | 0 (0.0%) | 12 (16.2%) | 3 (14.3%) | 4 (25.0%) | 10 (14.1%) | 1 (8.3%) |

***Table S8: Comparison between eating habits during the second lockdown and using food as a reward.***

| **Educational strategy** | **Food groups** | | | | | | | | | | | |
| --- | --- | --- | --- | --- | --- | --- | --- | --- | --- | --- | --- | --- |
| **Using food as a reward** | **Pandemic fruit consumption** | | | **Pandemic vegetable consumption** | | | **Pandemic legumes consumption** | | | **Pandemic whole-grain consumption** | | |
|  | **Less**  12 (12.1%) | **Not change**  65 (65.6%) | **More**  22 (22.2%) | **Less**  9 (9.1%) | **Not change**  71 (71.7%) | **More**  19 (19.2%) | **Less**  4 (4.1%) | **Not change**  74 (74.7%) | **More**  21 (21.2%) | **Less**  16 (16.2%) | **Not change**  71 (71.7%) | **More**  12  (12.1%) |
| **Completely disagree** | 4 (33.3%) | 29 (44.6%) | 7 (31.8%) | 4 (44.4%) | 30 (42.2%) | 6 (31.6%) | 2 (50.0%) | 33 (44.6%) | 5 (23.8%) | 6 (37.5%) | 33 (46.5%) | 1 (8.3%) |
| **Disagree** | 7 (58.3%) | 20 (30.8%) | 8 (36.4%) | 5 (55.5%) | 22 (31.0%) | 8 (42.1%) | 2 (50.0%) | 23 (31.1%) | 10 (47.6%) | 6 (37.5%) | 21 (29.6%) | 8 (66.7%) |
| **Neither agree nor disagree** | 0 (0.0%) | 11 (16.9%) | 3 (13.6%) | 0 (0.0%) | 10 (14.1%) | 4 (21.0%) | 0 (0.0%) | 11 (14.9%) | 3 (14.3%) | 2 (12.5%) | 10 (14.1%) | 2 (16.7%) |
| **Agree** | 1 (8.3%) | 5 (7.7%) | 4 (18.2%) | 0 (0.0%) | 9 (12.7%) | 1 (5.3%) | 0 (0.0%) | 7 (9.5%) | 3 (14.3%) | 2 (12.5%) | 7 (9.8%) | 1 (8.3%) |
| **Completely agree** | 0 (0.0%) | 0 (0.0%) | 0 (0.0%) | 0 (0.0%) | 0 (0.0%) | 0 (0.0%) | 0 (0.0%) | 0 (0.0%) | 0 (0.0%) | 0 (0.0%) | 0 (0.0%) | 0 (0.0%) |
